# Description of an Aneurism of the Arch of the Aorta Communicating with the Œsophagus

**Published:** 1817-11

**Authors:** M. Bricheteau


### *Description of an Aneurism of the Arch of the Aorta communicating with the Œsophagus.*

By M. Bricheteau, M. D.

Considerable lesions are very frequently developed in the secondary organs of the animal œconomy, without their existence being perceived by the individual who is the subject of them. Yet it is rare to observe the same phænomenon in those organs, the integrity of which is essential to the maintenance of life. Some examples of it have, however, been witnessed. That which I am now about to place upon record, is one of the most remarkable; and it exhibits, at the same time, a specimen of morbid anatomy, extremely curious and uncommon.

A man, aged about 50 years, was admitted into l'Hôtel-

Dieu, of Paris, on the 24th of February, 1816. When carefully examined on the morning visit, he exhibited no remarkable appearance. He was thin; and an expression of suffering was marked on his livid and dejected countenance. During the night, he was seized with a vomiting of blood, and suddenly expired. The body was conveyed to the amphitheatre of la Pitie; and the anatomical inspection of it there conducted by myself.

The exterior of the body was very pale; the abdomen excessively swollen. On opening it, I found the stomach uncommonly large, and distended with black blood, in which was a quantity of coagula. A little also was contained in the small intestines and cæcum. The thorax, on being exposed, presented, at the first glance, no organic change; but, on raising the heart from its site, I observed behind this organ, somewhat to the left of the vertebral column, an aneurismal sac, with thick and solid parietes, of an irregularly triangular form. It adhered to the vertebræ, which constituted its posterior boundary; was inseparably confounded by its right side with the left paries of the œsophagus, and intimately connected by its left with the trachea. This sac consisted in an aneurismal dilatation, which implicated at least six inches of the thoracic aorta, at its arch. The posterior paries having been destroyed by constant friction, it had contracted adhesions to the vertebral column. On detaching the sac with the scalpel, I found the bodies of four vertebræ completely exposed in it, hollowed out to the left, presenting a concave, rugged, and worm-eaten surface; while the sound intervertebral cartilages projected, of the natural colour, between them. The sac itself possessed only two sides, and measured four inches in its vertical, and three in its transverse, diameter. Its thickened parietes appeared generally to be of a fibrous nature; but they were, in several places, cartilaginous. Externally, they possessed the natural aspect of the aortic structure: internally, they were reddish, unequal, strewed with white and indurated points, and they did not seem susceptible of separation into layers. Towards the right, there existed a rounded aperture, of about one inch diameter in every direction, and communicating with the sac in the œsophagus, which, as before observed, was intimately connected with it. The circumference of this orifice was smooth, irregular, and of a fibrous nature. No trace of a recent rupture existed. It was almost completely obliterated by membraniform coagula of blood entangled in its margin, and which it required some force to tear away.

The history of aortic aneurism contains several cases, in which aneurismal tumours have burst into the trachea; but those, in which a rupture into the œsophagus has taken place from previous adhesion, are much more rare. *Bonetus* and *Morgagni* record no examples of the kind; nor have I found any in the Collection of *Lauth* on Aneurisms.* *Corvisart* says,† that an instance of it has been observed by *Dupuytren*, of which, however, no description is given.‡ The situation of the trachea, its stability, and the greater resistance of its parietes, compared to those of the œsophagus, satisfactorily explain this difference in frequency between the two terminations of aortic aneurism.

From the organic changes which its parietes had undergone, the condition of the vertebræ, and the singular aspect and configuration of the aperture of communication between the sac and the œsophagus, the aneurism, just described, must have, doubtless, very long existed. The orifice was not ruptured; and the inequalities which it presented, as well as other parts of its circumference, were smooth and well organized. The irruption of blood into the œsophagus had been prevented by the membranous and solid coagula, and the circulation carried on in this adventitious cavity, which represented a portion of the aorta. In proportion as the sac, adherent to the œsophagus, was worn away by the friction, coagula were probably deposited there, which consequently acquired adhesions to the surrounding parts, and served to prevent the escape of the blood into the œsophagus, until by some unknown cause the feeble barrier was broken down, which the lateral effort of the blood had not been able to destroy.

---

* *De Aneurismatibus Auctorum Latinorum Collectio.*

† *Essai sur les Maladies et les Lesions organiques du Cœur, &c.*

‡ *Sauvages* is the only writer whom I have consulted, who records a case of aortic aneurism communicating with the œsophagus. It bears a strong resemblance to that now under consideration. *Cadavere aperto*, says the author cited by Sauvages, *inveni ventriculum septem vel octo libris sanguinis distensum, aortam ad brachii magnitudinem per spatium septem vel octo pollicum dilatatam, et orificium denarii magnitudine aortæ et œsophago continuo commune, quod tamen quinque cristæ carneæ veluti valvulæ ex ambitu orificii oriundæ et circumpositæ potuerunt obturare; per hoc orificium sanguis ex aortâ fluxerat in œsophagum.* Nos. Meth, clas. ix. Hæmatemesis.

Verbrugge * asserts, that a similar obstacle long preserved the life of an individual suffering from aneurism, which communicated with the trachea. Every thing leads us to infer, that, in this case, the disease was marked by obscure symptoms; since M. Husson, a disciple of Corvisart, deeply versed in the diagnosis of lesions of the heart and large vessels, who examined the patient with great attention, only twelve hours before death, did not suspect its existence. This obscurity seems to depend on the situation of the disease, deeply developed between the vertebral column and the heart, and on the accidental disposition of the aneurismal sac. By these two peculiarities, the affection was rendered less severe in its consequences, and more difficult of appreciation by its symptoms. The caries of the bones, so remarkable in this case, was the result of reiterated friction, caused by the pulsations of the aneurismal tumour. It were useless, I think, to refute the opinion of those who have asserted that this phænomenon is produced by the action of the fluids contained in the aneurismal sac; since the cartilages, in this case, suffered no change, owing to their elastic property. Corvisart † has remarked, that moveable bones, such as the clavicle, are frequently displaced by the pulsations of the tumour. The excavation of bones, resulting from aneurism, are attributed by Scarpa ‡ to the action of the lymphatic system, which eats away the compressed parts, destitute of sufficient vitality whereby to resist its influence. He compares the ravages, thus exercised by the lymphatic vessels, to the devastation of worms on substances attacked by them. It sometimes happens, that the parietes of the sac wear away the contiguous bones, without themselves suffering extenuation. Verbrugge § ascribes this phænomenon to the activity of nutrition which exists in these parts, and rapidly repairs the losses which they have sustained ||

* *Op. citat.* † *Op. cit.*

‡ *Sull' Aneurisma Reflessioni et Osservazioni Anatomico-Chirurgiche.*

§ *Op. citat.*

|| *Bulletin de l'Athénée de Médecine de Paris.* Decembre, 1816.

PART

